# Altered Immune Cytokine Expression Associated with KoRV B Infection and Season in Captive Koalas

**DOI:** 10.1371/journal.pone.0163780

**Published:** 2016-10-05

**Authors:** Iona E. Maher, Damien P. Higgins

**Affiliations:** School of Life and Environmental Sciences, Faculty of Veterinary Science, the University of Sydney, NSW, Australia; Queensland University of Technology, AUSTRALIA

## Abstract

Koala (*Phascolarctos cinereus*) populations are increasingly vulnerable and one of the main threats is chlamydial infection. Koala retrovirus (KoRV) has been proposed as an underlying cause of the koala’s susceptibility to infection with *Chlamydia* and high rates of lymphoid neoplasia; however, the regionally ubiquitous, endogenous nature of this virus suggests that KoRV A infection is not sufficient for immune suppression to occur. A recently discovered exogenous variant of KoRV, KoRV B, has several structural elements that cause increased pathogenicity in related retroviruses and was associated with lymphoid neoplasia in one study. The present study assesses whether KoRV B infection is associated with alterations in immune function. Cytokine gene expression by mitogen stimulated lymphocytes of KoRV B positive (n = 5–6) and negative (n = 6–7) captive koalas was evaluated by qPCR four times (April 2014-February 2015) to control for seasonal variation. Key immune genes in the Th1 pathway (IFNγ, TNFα), Th2 pathway (IL 10, IL4, IL6) and Th17 pathway (IL17A), along with CD4:CD8 ratio, were assessed. KoRV B positive koalas showed significantly increased up-regulation of IL17A and IL10 in three out of four sampling periods and IFNγ, IL6, IL4 and TNFα in two out of four. IL17A is an immune marker for chlamydial pathogenesis in the koala; increased expression of IL17A in KoRV B positive koalas, and concurrent immune dysregulation, may explain the differences in susceptibility to chlamydial infection and severity of disease seen between individuals and populations. There was also marked seasonal variation in up-regulation for most of the cytokines and the CD4:CD8 ratio. The up-regulation in both Th1 and Th2 cytokines mirrors changes associated with immune dysregulation in humans and felids as a result of retroviral infections. This is the first report of altered immune expression in koalas infected by an exogenous variant of KoRV and also the first report of seasonal variation in cytokine up-regulation and CD4:CD8 ratio in marsupials.

## Introduction

Koala (*Phascolarctos cinereus*) populations are coming under increasing threat, and are now listed as vulnerable in all but the southern part of their range [1]. Threats include habitat loss, climate change, heatwaves, bushfires, motor vehicle accidents, dog attacks and disease [2–9]. Control of infectious disease, in particular infection with *Chlamydia* spp, is considered to be essential to the survival of some of the increasingly fragmented populations of koalas in northern Australia [10,11]. However, the impact of disease is variable across the koala’s range [12] and understanding drivers behind disease susceptibility is important in identifying and managing at-risk populations. Koala retrovirus (KoRV) has been proposed to be the underlying cause of the propensity of koalas to suffer from chlamydial disease [12,13], as well as the high rates of lymphoid neoplasia seen in koalas [13–16]. The koala retrovirus studied most extensively to date, KoRV A, has the unusual quality of being an endogenous retrovirus (i.e. integrated into the germline) in all populations studied north of the Victoria-New South Wales border [17], with open reading frames such that it is actively transcribed and all infected individuals are viremic [13,16]. The role of KoRV A in disease is uncertain due to a number of factors. Firstly, pathogenic endogenous retroviruses should be subject to negative selection and would not be expected to persist in the genome over the 22 200–49 900 years [18] that KoRV A has been endogenised in koalas, unless pathological effects were expressed intermittently or deleterious effects are counterbalanced by benefits [19]. Further to this, the majority of animals infected by KoRV A are apparently healthy and previous authors have suggested that cofactors (potentially including superinfection with exogenous pathogenic KoRV variants, recombination with other retroviruses, interactions with host haplotypes, interaction with somatic mutations or co-infection with other viruses such as herpesvirus) are likely to play a role in disease progression [16].

In recent years, a number of KoRV variants have been discovered [20–22]. All of these variants are presumed to be exogenous retroviruses due to incomplete penetrance in related family groups and a very low number of copies per cell in infected animals [20–23]. KoRV B is of particular interest as it contains several structural elements that are not present in KoRV A and have been associated with increased pathogenicity in other retroviruses [21]. These include duplicated enhancer regions in the long terminal repeat (LTR), which are associated with increased pathogenicity in the related FeLV [24], and a CETTG *env* protein motif, which, when attenuated, causes a reduction of cytopathic effects in the closely related GaLV [21,25]. KoRV B infection was also associated with an increased risk of lymphoid neoplasia in a small captive population [21]; further investigation is required to demonstrate causality however this suggests oncogenic potential of this new variant. To date, all koalas that have been identified with KoRV B infection are also infected with KoRV A [21], however, sampling has only been undertaken in areas where KoRV A infection is ubiquitous [26]. Both the severity of chlamydial disease and incidence of neoplasia have been related to KoRV A distribution, with much lower rates of overt clinical disease seen in Victorian and south Australian koala populations despite high rates of chlamydial infection [27,28] and much lower reported rates of lymphoid neoplasia reported in the same states [29]. While previous authors have attributed this to higher KoRV A incidence and viremic loads [29] the possibility of increased incidence of KoRV variants in areas where KoRV is endogenous having an influence on the pathogenicity of KoRV has also been proposed as an underlying cause of these differences [30].

Season is known to have a marked effect on immune function and cytokine expression in humans [31–33] and animals [34–36] and also has an effect on the CD4+ cell count and CD4:CD8 ratio in humans infected with HIV [37]. We know from previous studies that koalas have a seasonal variation in B and T cell lymphocyte composition [38] however the effect of season on cytokine levels or CD4:CD8 ratio has not been assessed. Our previous study [39] found no significant up-regulation in IL6; as this is known to have a seasonal variation in humans [40] we hypothesise that this may be due to the seasonal timing of blood collection.

The main aim of this study is to assess whether infection by KoRV B is associated with alterations in immune function. Cytokine gene expression by peripheral lymphocytes from KoRV B positive and negative koalas was evaluated by qPCR following mitogen stimulation. The secondary aim was to assess potential seasonal variations in cytokine levels in koalas and any potential seasonal effect of KoRV B infection. The koalas were sampled four times over the course of a year to examine this. As there is no precedent for this type of study in koalas, two climatic extremes were chosen (late summer and late winter) as well as two periods of changing day length (late spring and autumn) in order to increase the likelihood of detecting seasonal variations. We assessed key immune genes in the Th1 pathway (IFNγ, TNFα), Th2 pathway (IL 10, IL4, IL6) and Th17 pathway (IL17A), along with the ratio of CD4 to CD8 gene expression. Two different mitogen stimulation protocols, which have been previously shown to up regulate both Th1 and Th2 cytokines in koalas [39] were used over two time periods (5 and 12 hours). Five hour incubation has previously been shown to cause strong up-regulation of IFNγ and IL4 but only mild up-regulation of IL10 and no significant increase in IL6 in koalas [39]. Five hour and twelve hour stimulation were performed in order to maximise the potential for up-regulation to be seen in all cytokines evaluated.

## Materials and Methods

The study was carried out with the approval of the ethics committee of Taronga Conservation Society Australia/University of Sydney (permit no 4a/12/13) and the New South Wales Government (Scientific licence SL101220). All efforts were made to minimise animal suffering.

The study animals northern-type (not of Victorian origin) KoRV A positive koalas from a captive, *Chlamydia*-free collection, housed out-doors (Taronga Zoo 33.84°S, 154.24°E, Symbio Wildlife Park 34.20°S, 150.97°E) and with no history of disease in the 6 months prior to study commencement. Females that were pregnant or had pouch young (a joey under 7 months old) were not included. In each of four sampling events (April, August and December 2014 and February 2015) blood (5–10 ml) was collected from the cephalic vein of KoRV B negative (n = 7 April, n = 6 all other months) and KoRV B positive (August / December, n = 6; April / February, n = 5) koalas using manual restraint. Sex ratios were as follows: April 5 males, 7 females, August 4 males, 8 females December 5 males, 7 females and February 4 males, 7 females. Samples were collected from all individuals within a 1–2 week period during each sampling event. Due to the occurrence of pregnancy, lactation, and management decisions, the same individuals were not sampled at every time point, although most individuals were sampled multiple times; a total of 14 koalas (seven KoRV B positive and seven KoRV B negative) were included.

Immediately following collection, blood was placed into sodium heparin vials (Vacutainer; Becton Dickinson and Co, NJ, USA), and transported at room temperature for processing within 3 hours of collection. Peripheral blood mononuclear cells (PBMC) were separated using a percoll gradient [39]. Plasma was aspirated and stored at -80°C. Isolated PBMC were counted on an automated haematology analyser (Sysmex K4500: TOA Medical Electronics Co, Japan) and suspended at 1 x 10^6^ cells/ml in warm plain media comprising of RPMI and 10% FCS (F9423: Sigma Aldrich, Castle Hill, NSW, Australia). PBMC were then incubated in media containing either no additives (unstimulated); PMA 50 ng/ml and ionomycin 1 μg/ml (PMAio stimulation); or PMA 25 ng/ml and PHA 2 μg/ml (PMAPHA stimulation), as previously described [39]. Incubation was undertaken in a sealed tube at 37°C for two time periods: 5 hours and 12 hours. Cells were then harvested, washed twice in warm phosphate buffered saline, and resuspended in 1ml RNAlater (Applied Biosystems, Carlsbad, CA, USA). Cells were stored frozen at -20°C until RNA extraction.

The RNA was extracted using a RNAeasy minikit (Qiagen, Doncaster, VIC, Australia) following the manufacturer’s instructions. Samples were treated with amplification grade DNAse 1 (AMPDI-1KT; Sigma Aldrich, MO, USA) to remove possible contaminating DNA, then cDNA was synthetised using a Revertaid first strand cDNA synthesis kit (Thermo Scientific, Lithuania). To control for contamination with genomic DNA ‘no reverse transcriptase’ controls were made using the same protocol and omitting reverse transcriptase. The concentration and purity of RNA was assessed using a NanoDrop ND-1000 Spectrophotometer (Thermo Scientific, Wilmington, DE, USA).

### PCR

Reference gene (28s and GAPDH) and cytokine/CD (IL4, IL6, IL10, IFNγ, CD4 and CD8) qPCR were performed in triplicate as described previously [39]. Primers for TNFα and IL17A were sourced from the literature [41] and [42]. Conditions for these primers were re-optimised for use on the CFX96 Touch (Bio-Rad laboratories Inc., CA, USA).

KoRV A and KoRV B infection status was confirmed on entry to, and upon leaving, the study by RTPCR from cDNA from non-stimulated peripheral blood mononuclear cells (PBMC) samples, using generic KoRV, KoRV A and KoRV B *env* specific primers and probes sourced from the literature [21].

Viral RNA levels in cell free plasma were also assessed for KoRV A and B. Viral RNA was extracted using a viral RNA minikit (Qiagen, Doncaster, VIC, Australia) and cDNA synthesis was performed as above. A mosaic DNA KoRV plasmid was prepared by Integrated DNA technologies (Coralville, Iowa, USA) containing the KoRV A and B env genes. Standard curves were prepared for each run using a 10 fold dilution of this plasmid and the results for each run were normalised against the standard curve.

Each 20μL RTPCR reaction comprised: 10μL SensiFAST SYBR No-ROX, (Bio-line, London, UK), 0.9μl 50 μM primers (F and R), 0.625μl TaqMan probe, 200ng of template cDNA, and PCR grade water. Cycling conditions were: 95°C for 5 min, followed by 45 cycles of 95°C for 10 s and 62°C for 20 s, on a CFX96 Touch real-time thermocycler (Bio-Rad laboratories Inc., CA, USA). Positive and negative controls were performed in each PCR run using plasmids containing synthetic KoRV A and B sequences and water, respectively.

### Statistical analysis

Raw Cq values were transformed to 2^-*Cq*^ for analysis. Baseline gene levels in un-stimulated samples were measured using the 2^-ΔCq^ method [43] and up-regulation of cytokine expression (fold change) was assessed using the 2^-ΔΔCq^ method, where ΔΔCq = (Cq of target—Cq of reference gene) at any time point—(Cq of target—Cq of reference gene) at time zero hour [44]. Cytokine expression was normalised against the geometric mean of the two reference genes 28s and GAPDH. These have previously been identified as valid reference genes for mitogen stimulation of koala PBMC’s [39]. If Cq values fell below the limit of quantification of the assay they were excluded from analysis.

To examine the combined effect of KoRV B status on cytokine up-regulation we constructed partial least squares discriminant analysis (PLS-DA) models for the cytokine up-regulation data, using a publicly available package (plsreg1) in R (https://www.r-project.org/). PLS-DA models were built after natural log transformation, followed by unit-variance scaling and mean centering of the data. Predictive power was assessed by computing the Q^2 statistic by leave-one-out cross validation.

Further analysis was performed to explore individual differences in the data. The data were assessed for normality using a Shapiro-Wilk analysis and, as none of the data were normally distributed and data sets could not be consistently transformed to normality, non-parametric analyses were performed. Mann Whitney U tests were used for 2 sample non-parametric comparisons (e.g. between KoRV B positive and negative groups, 5h and 12h stimulations, PMAio and PMAPHA stimulations and sex groups) and Kruskal Wallis one-way ANOVA tests were used for comparison between more than two groups (e.g. across seasons). These statistical analyses were performed using GenStat 17^th^ edition (VSN International Ltd., Hemel Hempstead, UK). A *p* value of <0.05 was considered significant.

## Results

### Differences between 5h and 12h stimulation and mitogen protocols

An increase in incubation time (from 5 to 12 hours) was usually not associated with increased up-regulation for the majority of cytokines. The level of expression was sometimes significantly decreased with an increase in incubation time (TNFα (December PMAio *p = 0*.*008*) and IL10 (August PMAio *p <0*.*001*, December PMAPHA *p = 0*.*02*)). An increase in cytokine expression with increasing incubation time was seen only once (TNFα August PMAPHA *p <0*.*001*). As no overall significant difference in up-regulation was found, the 12 hour stimulation was not performed for the February sampling.

PMA ionomycin was the most effective agent for up-regulation of IFNγ (this was significantly different in April *p = 0*.*007*, August *p = 0*.*006*, February *p = 0*.*003* (5 hour), August *p = 0*.*004*, December *p = 0*.*032*, (12 hour)), IL10 (significantly different in August *p = 0*.*01* (5 hour)) and IL4 (significantly different in February *p = 0*.*029* (5 hour)). Conversely, IL6 was generally more up-regulated with the PMAPHA protocol compared to the PMAio; significantly so in December (*p = 0*.*001*) and February (*p = 0*.*007*) (both 5 hour). Both protocols caused up-regulation of IL17A and TNFα and there was no significant difference between them.

### Seasonal variation in cytokine expression

**Fig 1:**
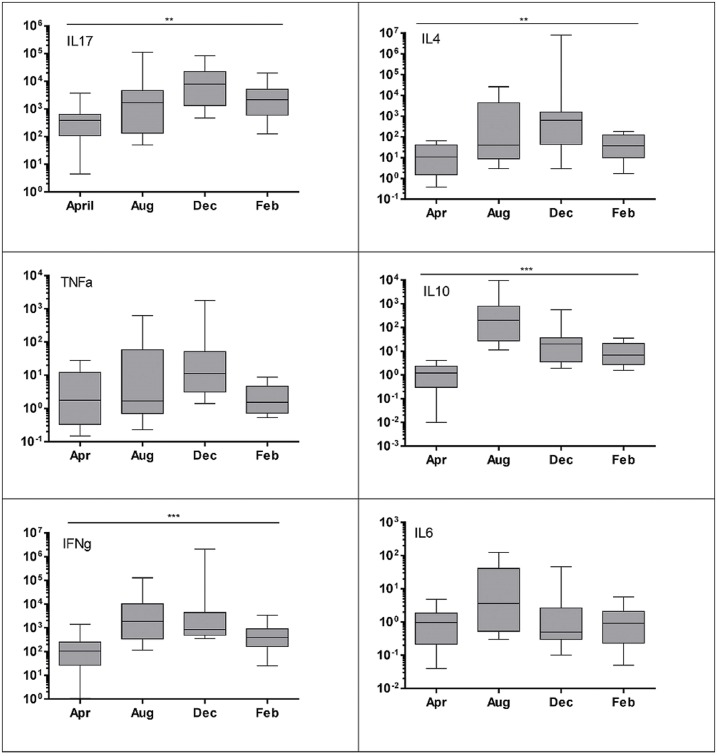
Seasonal difference in cytokine up regulation (PMAio 5h stimulation). * p < 0.*05*, **p < 0.*01*, ***p < 0.*001*. Kruskal-Wallis one-way ANOVA. Results are expressed as fold change calculated using the ΔΔCq method [44]. Box represents the 25^th^ to 75^th^ percentiles, middle line is median and whiskers are the minimum to maximum values.

There was a marked seasonal difference in mitogen stimulated up-regulation of most of the cytokines. Fig 1 illustrates the differences for the PMAio 5 hour stimulation although these seasonal changes were consistent across mitogen stimulation protocols and were seen in KoRV B positive and negative animals (see Fig 1). Interleukin 17A (*p = 0*.*007*) and IL4 (*p = 0*.*009*) were significantly more upregulated in the December sampling while TNFα approached significance (*p = 0*.*066*). Interleukin 10 (*p <0*.*001*) and IFNγ (*p <0*.*001*) were more strongly up-regulated in August than in other seasons. Interleukin 6 was also more upregulated in August but this difference was not significant (see Fig 1).

**Fig 2:**
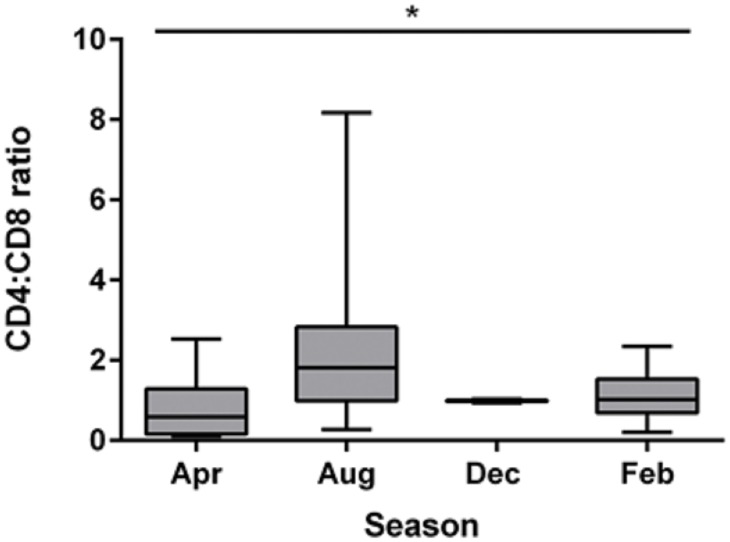
Seasonal difference in CD4:CD8 ratio. * *p < 0*.*05* Kruskal-Wallis one-way ANOVA. Ratio calculated after CD4 and CD8 results were calculated using the ΔCq method [43]. Box represents the 25^th^ to 75^th^ percentiles, middle line is median and whiskers are the minimum to maximum values.

**Fig 3:**
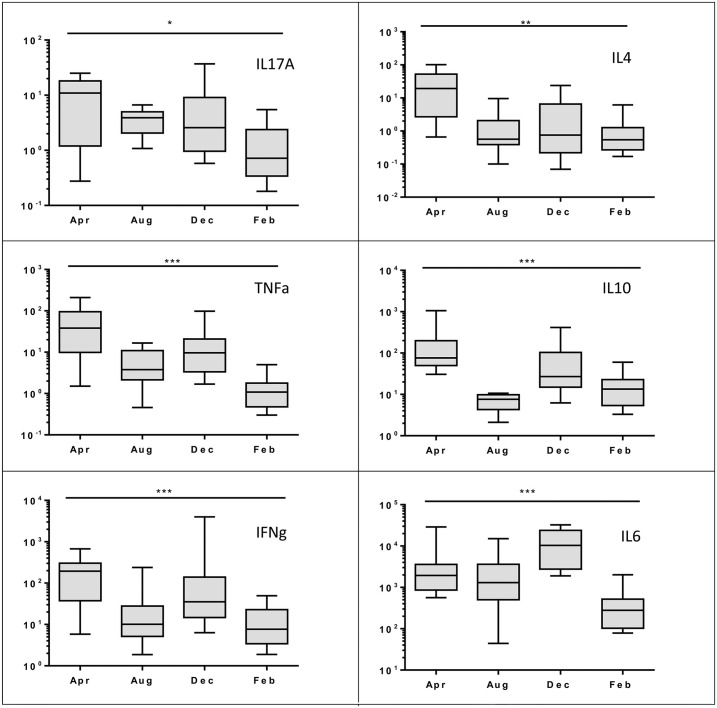
Seasonal difference in baseline cytokine levels. * *p < 0*.*05*, **p < 0.*01*, *** p < 0.*001* Kruskal-Wallis one-way ANOVA. Results are relative expression of cytokine levels compared to reference genes calculated using the ΔCq method [43]. Box represents the 25^th^ to 75^th^ percentiles, middle line is median and whiskers are the minimum to maximum values.

There was a significant seasonal variation in the CD4:CD8 gene expression ratio (*p = 0*.*021*), which was highest in August and lowest in April (see Fig 2), and there was a seasonal variation in the baseline production of all cytokines. Some of the patterns seen in the baseline variations were similar to those in the stimulated cells with IL17A *(p = 0*.*014*), TNFα (*p <0*.*001*) and IL4 (*p = 0*.*002*) all higher in April and December and lower in February and August. In contrast IL10 (*p <0*.*001*) and IFNγ (*p <0*.*001*) baseline expression differed to the stimulated cells being higher in April and December and lower in February and August, and IL6 (*p <0*.*001*) was highest in December compared to the maximum up-regulation which was seen in August (see Fig 3).

### Differences in up-regulation between KoRV B positive and negative groups by season

**Fig 4:**
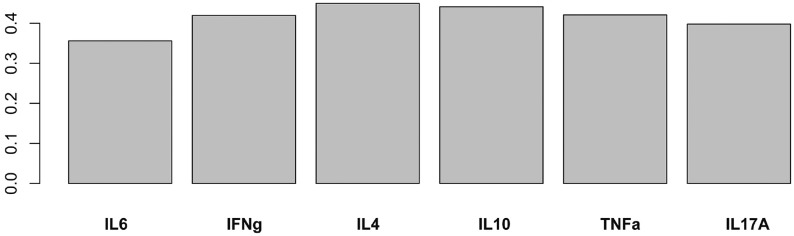
Relative contribution of cytokine up-regulation to KoRV B predictive relationship in December.

To investigate the combined effect of KoRV B status on the (log transformed) cytokine up-regulation profiles, we built a 1-component PLS-DA for the four sampling periods however predictive relationships were only found in the December sampling. In December the Q2 (Q-squared) value was 0.13, which is the predicted residual sum of squares, a measure of how good the model is at predicting new observations, with values of Q2 between 0–0.5 indicating a weakly predictive relationship between KoRV B status and gene up-regulation profiles. Further loadings analysis showed that while all cytokines contributed to the predictive relationship with KoRV B positive status the largest contributions were from IL10 and IL4 (see Fig 4)

**Fig 5:**
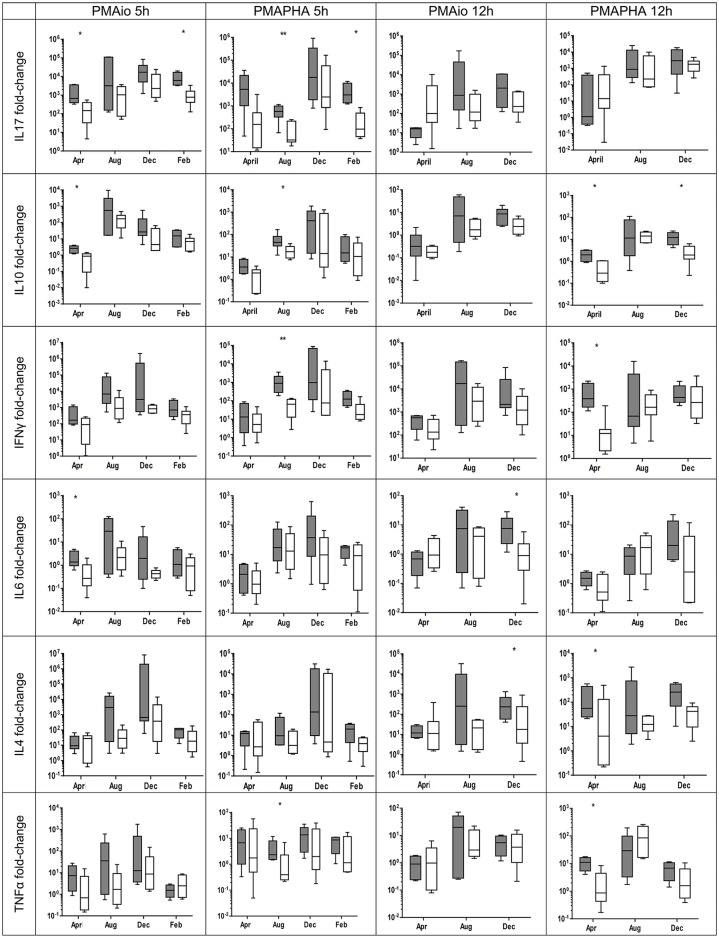
Cytokine fold change in KoRV B positive and negative groups by collection period. * p < 0.*05*, **p < 0.*01*, ***p < 0.*001* Mann Whitney U. White box is KoRV B negative and grey box is KoRV B positive. Results are expressed as fold change calculated using the ΔΔCq method [44]. Box represents the 25^th^ to 75^th^ percentiles, middle line is median and whiskers are the minimum to maximum values.

When examined on individual basis significant differences in cytokine up-regulation were found between KoRV B positive and negative groups over both mitogen stimulation protocols, both incubation periods and all collection periods (see Fig 5). In every instance of significant differences between groups, the KoRV B positive group always showed greater up-regulation than the KoRV B negative group.

The KoRV B positive group showed significantly greater up-regulation in three out of four sampling periods for IL17A and IL10 (see Fig 5) and two out of three for IFNγ, IL6, IL4 and TNFα (see Fig 5). On several occasions, differences in IFNγ up-regulation between groups approached significance (PMAio 5h August *p = 0*.*066*, PMAPHA 5h February *p = 0*.*067*).

There was no significant effect of sex on cytokine expression in any of the time periods.

### Viral load, KoRV status and cytokine levels

KoRV A viral loads varied from 1.8 x10^3^ to 6.1 x10^6^ copies per ml. There was no significant difference between KoRV A viral loads in KoRV B positive and negative koalas. KoRV B titres were much lower (4.2 x10^2^ to 6.0 x10^3^ copies per ml) and were in some cases undetectable in koalas that tested KoRV B positive on cDNA. There was no relationship between the up-regulation of any of the cytokines, the CD4:CD8 ratio or sex with the KoRV A or B viral loads.

### Differences between KoRV B positive and negative groups in baseline cytokine expression and CD4/CD8 ratio

**Fig 6:**
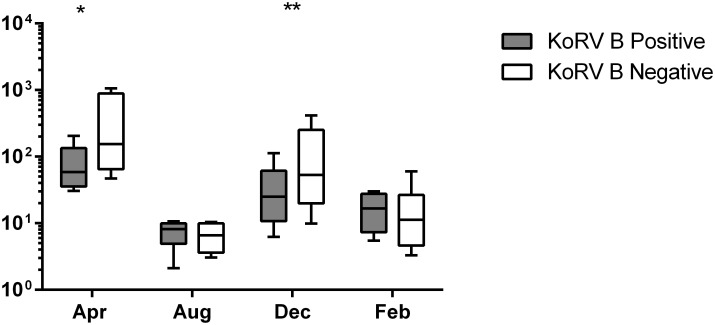
IL10 baseline production in KoRV B positive and negative groups. * p < 0.*05*, **p < 0.*01* Mann Whitney U. Results are relative expression of cytokine levels compared to reference genes calculated using the ΔCq method [43]. Box represents the 25^th^ to 75^th^ percentiles, middle line is median and whiskers are the minimum to maximum values.

The only cytokine that displayed any significant differences in baseline expression was IL10; this was significantly higher in the KoRV B negative group in two sampling periods (April (*p = 0*.*01*) and December (*p = 0*.*003*)) (see Fig 6).

There was no difference between KoRV B positive and negative groups in CD4:CD8 in any of the sampling periods and there were no differences detected between sexes.

## Discussion

This study reveals that, among captive, KoRV A-positive koalas in temperate latitudes, significant changes to lymphocyte composition and cytokine expression are associated with KoRV B infection and with season. Varying degrees of up-regulation were seen across the Th1 pathway (IFNγ, TNFα), the Th2 pathway (IL 10, IL4 and IL6) and the Th17 pathway (IL17A) and, across seasons, CD4:CD8 expression profiles also varied significantly.

One of the most consistent findings in this study is the up-regulation of IL17A in KoRV B-infected koalas. This is significant as previous studies have found that IL17A is an immune marker for chlamydial disease severity and pathogenesis in koalas [42] and, in the murine model, the magnitude and duration of infection with *C*. *muridarum* is significantly decreased in the absence of IL17 [45]. If KoRV B positive koalas exhibit a greater Th17 immune response when challenged with chlamydial infection this could result in increased susceptibility to chlamydial infection and an increase in disease severity. Comparing this result with the relationship between IL17 cells and other retroviral infections is difficult. Although this finding contrasts with the marked decrease in IL17 producing cells seen in early HIV infection [46,47] it is likely that all of our koalas are chronically infected, and the relationship between chronic HIV infection and Th17 cells is still unclear [48]. Additionally, HIV and KoRV utilize different cell binding and entry mechanisms [25,49].

The PLS-DA model was only predictive of an effect of KoRV B status on gene up-regulation in the December sampling, which may reflect the maximum up-regulation of a number of cytokines in this sampling period including IL17A, IFNγ, TNFα and IL4. In this sampling period all of the cytokines measured contributed to the relationship between KoRV B status and cytokine up-regulation although the largest contribution was from the Th2 cytokines IL4 and IL10. While this could suggest a slight Th2 bias during this period there was also a significant contribution from Th1 cytokines (IFNγ, TNFα) and Th17 cytokines (IL17A). In the three other sampling periods, although there was no overall predictive relationship between KoRV B status and cytokine up-regulation significant differences were frequently found, most consistently for IL17 and also for both Th1 and Th2 cytokines. This lack of statistically significant up-regulation may reflect the small sample size, which was necessitated by the small pool of KoRV B tested koalas which was available for sampling. In many instances, where there was no significant difference between KoRV A and B groups there is a trend for higher cytokine up-regulation in KoRV B koalas. This can be seen for example with IFNγ in August and December; KoRV B koalas are frequently more up-regulated but the difference only significant once. On every occasion where a significant difference between groups exists KoRV B is always higher which strongly suggests these changes are not due to random variation. Overall, rather than a clear Th1 or Th2 pattern there appears to be general up-regulation of all cytokines measured in KoRV B infected koalas. This effect may be strongest in the time of year when mitogen responses are strongest (December) but is also seen through the rest of the year.

An effective cell mediated immune response is essential for the defence against intracellular pathogens such as *Chlamydia* spp [50–52]. Resistance to chlamydial infection is associated with increases in Th1 cytokines, such as IFNγ, which promote cytotoxic T cell responses [51]; conversely, a Th2-dominated response, characterised by increased IL4 and promoted by IL10, is associated with persistence of infection. These Th2 responses inhibit Th1 responses, promote antibody responses that are less able to eliminate intracellular forms of *Chlamydia* spp and promote structural damage as a result of fibrosis [53]. A Th1 to Th2 shift has been proposed to play an essential role in the immunosuppression caused by HIV [54] and thus the role of retroviral infection in altering cell mediated immunity and increasing susceptibility to chlamydial infection has long been suspected. More recent work in retroviral infections in both humans (HIV) and felids (FIV, FeLV) have not supported a clear Th1 to Th2 shift, and increases in both Th1 and Th2 cytokines were found in these infections [55–57]. It has been proposed that rather than a clear Th1 to Th2 shift, retrovirus infection induces cytokine deregulation and alterations in cytokine transcription, which result in inadequate innate and cell mediated immune response to other pathogens [58]. The up-regulation in both Th1 and Th2 cytokines in KoRV B infected koalas found in this study is consistent with this, and if immune dysregulation is occurring in KoRV B positive koalas, this could alter cell mediated immunity and increase susceptibility to chlamydial infection.

Additionally, immune dysregulation in KoRV B-infected koalas may have consequences for more than susceptibility to *Chlamydia* spp. An essential role of immune surveillance is removal of early neoplastic cells, and immune dysregulation secondary to retroviral infection is a well-known cause of neoplasia in humans [59]. If the changes seen in this study are sufficient to cause dysregulation *in-vivo*, this might contribute to the association with lymphoid malignancies seen in the initial study population [21] and also the high levels of lymphoid neoplasia reported in both wild [15] and captive populations in Australia [16].

There are several potential mechanisms by which KoRV B could induce immune dysregulation. As previously mentioned, KoRV B has several structural elements that are associated with increased pathogenesis in other retroviruses [21]. KoRV B also contains the immunosuppressive domain p15E [21] which is identical to that found in other related retroviruses (FeLV, GaLV, PERV) and causes a multitude of immunosuppressive effects (reviewed in [60]). While this is also present in KoRV A [16] there is some evidence that protein structural changes may be present in the p15E sequence found in endogenous KoRV A [61], which may result in differences in immune effects of KoRV A and KoRV B.

It is also possible that KoRV B (along with other variants) may provide a means of mobilizing endogenous KoRV A by circumventing the super-infection block present in koalas where KoRV A exists as an endogenous virus [22]. A recent study found that all 39 KoRV A proviral loci identified in the sire and dam of a northern Australian koala were vertically transmitted to the progeny, and no new proviral loci were identified [18]. This absence of new proviral integrants suggests superinfection by endogenous KoRV A is unlikely (at least in these individuals). Retroviral superinfection resistance is an interference mechanism, established after primary infection, that prevents the cell being infected by a similar type of virus (reviewed in [62]). Super-infection with HIV in humans has been associated with the generation of recombinant HIV strains and possibly increased disease progression [62] and this may also be occurring with KoRV.

No relationship was found between plasma viral load of KoRV A or B and cytokine expression or CD4:CD8 in this study. Viral load is a well known marker of retroviral disease progression in humans [63] and cats [64]. Previous studies have found an increased viral load in koalas with lymphoid neoplasia [13] and the authors suggested a causal link between KoRV infection and neoplasia, however the number of neoplasia affected koalas was low (n = 4) and this same study found no significant relationship between viral load and chlamydial disease. All of the koalas in our study were healthy with no evidence of immune suppression or neoplasia and so it may be unsurprising no relationship was found with viral load. Further research into viral loads in koalas presenting with symptoms of immune suppression compared to healthy koalas will be of great interest.

An increase from 5 hour to 12 hour stimulation was not generally associated with any increased up-regulation of cytokine production, which is likely to reflect that maximal mitogen stimulation with PMAio and PMAPHA requires only a short period of time in koalas. Additionally, longer time periods could lead to cell apoptosis as expression of some cytokines (IL10 and TNFα) decreased with 12 hour stimulation. This is in contrast to other studies where koala PBMC’s were stimulated with inactivated *C*. *pecorum* and up-regulation peaked at 24 hours stimulation [41]. This difference is likely to reflect the inherent ability and time needed for activated mitogens verses antigens to stimulate cells. In our previous study, no significant up-regulation of IL6 was found with any protocol [39] which was thought to be due to insufficient stimulation period. In this study we found, rather than increased production with longer incubation, the expression of IL6 is very seasonal, with up-regulation seen in August and December and very little in April and February.

Given the results of the present study, it will be important to conduct epidemiological surveys to determine prevalence and transmission dynamics of KoRV B. Since its initial discovery in the Los Angeles Zoo population [21] KoRV B has been reported in 2 European Zoos [65] and in wild populations in Northern Australia [23,66], however the prevalence of KoRV B in koalas in Australia is currently unknown. Given that both wild and captive animals in unrelated and geographically separated populations have tested positive it is likely that this variant is relatively widespread, at least in northern Australia.

KoRV B and KoRV J, a variant of KoRV that was discovered in koalas in Japanese zoos [20], are sufficiently similar to be considered in the same sub-group [67]. KoRV J was detected in 67.5% of Queensland origin koalas, but in none of the Victorian origin koalas tested [20]. It would be of interest to determine the distribution and prevalence of these variants nationally, as several researchers have reported low rates of clinical disease in Victoria, despite high rates of chlamydial infection [27,28] and, although generic variation in chlamydial strains is likely to be a important element of this, the absence of pathogenic KoRV variants has also been suggested as a possible explanation [30].

The seasonal variation detected in the present study echoes that seen in studies of humans [31] and is of relevance to both design of future studies, and the potential for seasonal fluctuations in disease susceptibility. Specifically, studies looking at mitogen stimulation on human PBMC’s have found greater up-regulation of IFNγ, IL10 and IL6 in winter compared to summer [33], and greater up-regulation of IL10 in winter and spring [32] which is comparable to our finding of up-regulation of IL10 and IFNγ and a trend towards IL6 up-regulation in August.

Very few studies have investigated seasonal variation in mitogen-stimulated cytokine expression profiles in wildlife and, to the authors’ knowledge, this is the first paper to examine this in any marsupial. The significance of these seasonal variations is manifold. If this pattern is seen in other populations then it will confound interpretation of any differences observed in cytokine expression between populations if samples are collected in different time periods or geographical areas. Additionally, if researchers are interested in investigating particular cytokines such as IL6 (which we have previously found very low levels of up-regulation [39]) or IL10 it may be worth sampling animals over the winter for maximum possible cytokine response to mitogen stimulation. The mechanisms underlying these seasonal variations are unknown. Seasonal variation in cytokine expression in humans is frequently linked to UV exposure and vitamin D levels [31,33]. In koalas, UV exposure is essential for normal bone development [68,69] and, while this may be an important variable, other seasonal factors such as stress [70], an increase in toxic plant metabolites in the diet [71], photoperiod [72], fluctuation in nutrient availability [73] or hormonal fluctuations [72] might also play an important role.

The CD4:CD8 ratio was lowest in April and highest in August, consistent with patterns found in humans [40]. This seasonal variation in CD4 and CD8 lymphocyte populations aligns with previous marked seasonal alterations in B and T cell populations found in koalas [38]. The CD4:CD8 ratio is often used as a measure of immune suppression in retrovirus infected individuals including HIV and FIV [74–76] however this wide seasonal variation in healthy animals indicates it should be used with caution to investigate immunosuppression in koalas.

This study has demonstrated significant and consistent differences in lymphocyte responses of koalas infected with KoRV B, compared to those not infected. The presence of a strong affect of KoRV B on Th17 response, and evidence for broader immune dysregulation, suggests that these animals may be more prone to chlamydial disease and possibly lymphoid neoplasia. Chlamydial disease is the most significant disease threat affecting wild koalas [2,27,77] and the presence of this new variant may go some way to explaining the differences in susceptibility to *Chlamydia* spp and severity of disease seen between individuals and populations. More comprehensive epidemiological and immunological studies into virus distribution, transmission and pathogenesis are needed to further elucidate this virus’ role in pathogenesis, and inform management of captive and free-ranging populations. The present study has provided a unique insight into the immune effect of a newly discovered variant of KoRV. Further studies into KoRV and its effect on the immune system of koalas are essential to understand its place among the forces that drive infectious disease and neoplasia in this species. Such knowledge will aid management practices in captive and wild populations and will aid in the preservation of this iconic Australian marsupial.

## Supporting Information

S1 File Cytokine ΔΔCq values by collection period and mitogen stimulation protocol. (XLSX)

S2 File CD4:CD8 ratios by collection period. (XLSX)
